# An Egg Volume Measurement System Based on the Microsoft Kinect

**DOI:** 10.3390/s18082454

**Published:** 2018-07-28

**Authors:** Ting On Chan, Derek D. Lichti, Adam Jahraus, Hooman Esfandiari, Herve Lahamy, Jeremy Steward, Matthew Glanzer

**Affiliations:** 1 Department of Geomatics Engineering, University of Calgary, 2500 University Dr NW, Calgary, AB T2N 1N4, Canada; ddlichti@ucalgary.ca (D.D.L.); avjahrau@ucalgary.ca (A.J.); stewardj@ucalgary.ca (J.S.); 2 School of Geography and Planning, Sun Yat-sen University, Guangzhou 510275, China; 3 Department of Mechanical Engineering, University of British Columbia, Vancouver, BC V6T 1Z4, Canada; hooman.esfandiari@ubc.ca; 4 Saskatchewan Polytechnic, Moose Jaw Campus, SK S6H 4R4, Canada; Herve.Lahamy@saskpolytech.ca; 5 Skytech Solutions Ltd., Calgary, AB T2L 1Y4, Canada; m.glanzer@skytechsolutions.ca

**Keywords:** 3D reconstruction, geometric modelling, volume estimation, 3D range camera, feature recognition, image processing

## Abstract

Measuring the volume of bird eggs is a very important task for the poultry industry and ornithological research due to the high revenue generated by the industry. In this paper, we describe a prototype of a new metrological system comprising a 3D range camera, Microsoft Kinect (Version 2) and a point cloud post-processing algorithm for the estimation of the egg volume. The system calculates the egg volume directly from the egg shape parameters estimated from the least-squares method in which the point clouds of eggs captured by the Kinect are fitted to novel geometric models of an egg in a 3D space. Using the models, the shape parameters of an egg are estimated along with the egg’s position and orientation simultaneously under the least-squares criterion. Four sets of experiments were performed to verify the functionality and the performance of the system, while volumes estimated from the conventional water displacement method and the point cloud captured by a survey-grade laser scanner serve as references. The results suggest that the method is straightforward, feasible and reliable with an average egg volume estimation accuracy 93.3% when compared to the reference volumes. As a prototype, the software part of the system was implemented in a post-processing mode. However, as the proposed processing techniques is computationally efficient, the prototype can be readily transformed into a real-time egg volume system.

## 1. Introduction

The chicken egg industry is historically an important asset to many countries. In Canada, there were more than 1000 registered egg farms in 2015, generating more than 960 million Canadian dollars of cash receipts in total [1]. Among all the essential procedures within the industry, egg volume measurement plays a very important part as it is directly related to its product quality and revenue. Additionally, egg volumes of some birds are also of high interest for ornithologists, ecologists, and climatologist as it is closely related to the global warming issues [2,3,4]. Therefore, a user-friendly and cost-effective egg volume measurement system is always desired for many individuals to obtain accurate egg volume quickly, and without directly contacting the eggs.

Currently in the Canadian egg industry, the egg volume estimation is mostly performed using contact-based weighting systems. This method can lead to problems of cracking or breaking of egg shells. The egg volume estimation problem has been studied by many researchers for decades, e.g., [5,6,7]. Thanks to the advancement and declining cost of digital imaging sensors, much focus is being put on non-contact measurement methods for egg volume/size measurement. For examples, Bridge et al. [8] proposed a digital camera-based method in which the egg was manually selected from the digital images by the operator. More than 84% of their testing samples had less than 2% of volume error. This method did not involve any mathematical formulation of egg curve, instead, it broke the egg image into many horizontal pixel slices and estimated the volume of each slice with the volume formula of a circular cylinder. Zhou et al. [9] presented an image-based method in which a specific stage was created to hold a levelled egg under lighting so the shadow of the egg could be captured using a digital camera. Subsequently, the length and the breadth of the egg could be derived. The derived quantities were then substituted into an egg volume equation proposed by [10] to estimate the volume. The paper reported that approximately 90% of their measurement resulted in 2 mL of volume error. However, this method requires manual placement of the egg onto the system’s stage with an egg shape hole, and it cannot be automated easily. Also, it was not guaranteed that the egg would have been perfectly levelled. Troscianko [11] proposed a two-dimensional (2D) formula for egg shape, and manually extracted egg edge points from digital images taken with gridded background to estimate the egg size and volume. This method claimed to have very low volume error (<0.74%) but it required several manual steps to measure the egg dimensions on the top of a paper grid with a digital camera. Additionally, lens distortions were not considered in this technique. 

Zalhan et al. [12] developed an egg radius measurement prototype with higher than 96% of volume accuracy using a digital camera. The egg was vertically placed on a stage and the camera was aligned with the egg’s zenith. The pixel size of the egg images was estimated from reference values obtained from a coordinate measuring machine (CMM). The CMM is often a costly procedure with high levels of operational complexity. A more user-friendly egg geometry measurement method is proposed by [13]. The authors used a smartphone camera to classify eggs into different grades with different size ranges, subsequently, the reported classification accuracy was higher than 80% using this technique. Their method required a coin with known size being placed next to the egg as a reference object. By counting the number of pixel after detecting the coin from the image, the size of the egg can be estimated. The egg pixels were modelled as an ellipse for size estimation purposes, and camera lens distortion was not considered. These conditions had limited the egg size estimation accuracy.

Zhang et al. [14] performed a photogrammetric reconstruction of the egg placed inside a target field, they then used a convex hull algorithm to estimate the volume of the reconstructed three-dimensional (3D) egg. They also used the Monte Carlo method to estimate a volume calibration factor with some reference egg volumes to refine the volume estimation that could achieve 99% accuracy. Although this method is accurate, several images must be taken each from a different position and orientation to be used in the photogrammetric bundle adjustment process. In addition and as an auxiliary limitation, a target field is needed to provide tie points between the images.

Excluding the method involving the photogrammetric process [14], the aforementioned techniques mainly used digital 2D images, and therefore only incomplete 3D egg geometry could be recovered. However, the photogrammetric process requires several images of an egg taken under specific conditions to satisfy the bundle adjustment requirements. This may require some operator training and it is not often time-efficient. On the other hand, the image-based techniques often require accurate external size references or target field. To overcome those drawbacks, as an alternative of 2D images one can propose using 3D imaging to directly capture the egg geometry without the use of any external references, hence estimate the volume directly from the raw measurement. In this paper, we use a popular cost-efficient 3D camera, the Microsoft Kinect 2.0 (~$200 USD), to develop an egg volume measurement system prototype with a custom-developed egg geometric models and the corresponding point cloud processing algorithm. Since only one static Kinect is involved, approximately half of the egg shell can be captured. Therefore, common volume estimation mesh-based techniques such as convex hull or alpha shape [15] cannot be used to accurately estimate the volume. Instead, we have developed a set of novel 3D egg geometric models, through which the egg shape parameters can be accurately estimated based on the least-squares method only capturing part (roughly one half) of the egg surface. The main scientific contribution of this research is that the proposed 3D models of eggs along with the processing algorithm allows individuals to build up a simple and cost-efficient egg metrological system with only one single Kinect (or other similar sensors) without resort to setting up any target fields or performing registration processes. As hand-held and smart phone-based scanners become available to users, the proposed algorithm is compact enough to be implemented on those platforms to support on-line measurement application. The paper is organized as follows: Section 2 focuses on the system design prototype including the proposed geometric models of an egg, and the associated egg point cloud processing algorithm; Section 3 gives descriptions of the experiment, the datasets and the assumptions; Section 4 and Section 5 are the experimental result analysis and conclusion, respectively.

## 2. System Design Prototype, Mathematical Models and Algorithm

### 2.1. Proposed Measurement System Overview

**Figure 1:**
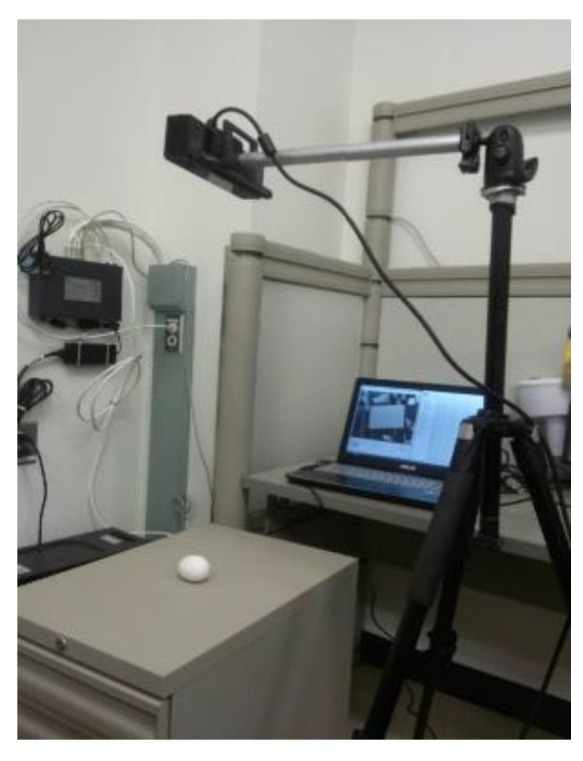
Proposed System Design Prototype (Photo taken at the University of Calgary’s Image Metrology Lab).

As seen in Figure 1, the measurement prototype consists of a Microsoft Kinect 2.0 3D camera, a camera tripod, a stage that is at the nadir of the camera and a laptop computer. In the following subsections, we focus on details and application examples of the Kinect, the proposed geometric model of the egg, and the associated egg processing algorithms, which all contribute to the entire system prototype.

### 2.2. Microsoft Kinect 2.0

The Microsoft Kinect was originally designed for detecting human motion in real-time for the dedicated video game console, Xbox. Later, a software development kit (SDK) was also provided by the manufacturer [16] to help users develop their specific applications which involve 3D short-range sensing. To-date, there are mainly two major versions of the Kinect: Kinect 1.X (1.0, 1.5-8) and Kinect 2.0. The 3D data collection principles of the two versions are significantly different. The Kinect 1.0 is equipped with an infrared emitter and sensor distributed close to both ends of its physical body. The emitter, sensor, and the target form a triangle in the object space so the target position can be readily estimated by the triangulation principle. On the other hand, the Kinect 2.0 uses the time-of-flight (TofF) [17] technique to estimate target positions. The embedded infrared sensor emits signals and senses the returned signals after hitting a target. The target position can be then derived from the observed phase shift between the emitting and returning signals, with the known speed of the light. Considering the improved hardware of Kinect 2.0, and higher accuracy, typically less than 2 mm within 2 m object-to-sensor distance [18], the Kinect 2.0 was used in this work. There are many application examples of the Kinect 2.0: biomedical engineering [19,20,21]; indoor mapping [22] and structural deformation measurement [23].

For the Kinect 2.0, the infrared camera is already pre-registered to a Kinect 3D image space with meter as the unit so each pixel of the infrared camera is associated with a 3D coordinate in the Kinect 3D image space. According to [24], the origin of the Kinect 3D image space lies on the front plate, with approximately 8 cm from left surface and 2.2 cm from the top surface. At each frame, the raw observation of Kinect 2.0 includes a depth image (424 × 512 pixels), an infrared image (424 × 512 pixels) and an RGB image (1080 × 1920 pixels). The depth image can be considered as a gridded point cloud while the returned infrared light intensity is stored in the infrared image. Only the depth and infrared images of the Kinect 2.0 are used for this work, as the RGB image is not registered with the infrared camera by the manufacturer.

### 2.3. Geometric Models of the Egg Shell

#### 2.3.1. 2D Egg Models

**Figure 2:**
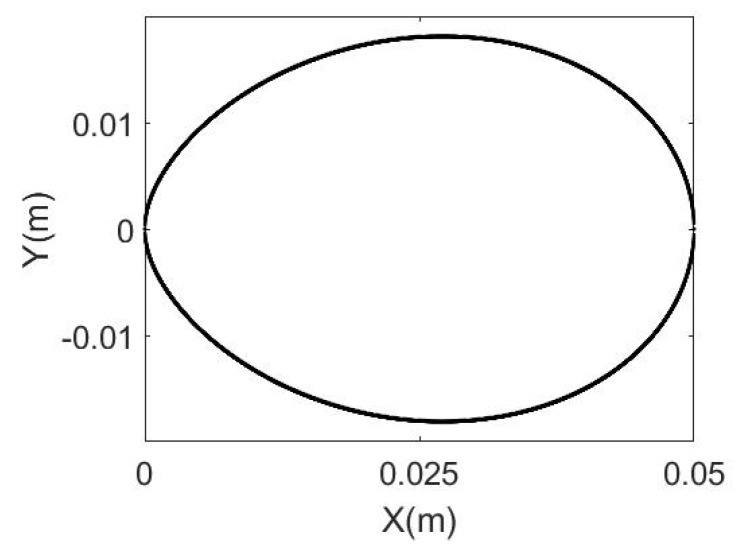
Two-dimensional Yamamoto (2017)’s Egg Curve.

A 2D egg curve equation normally represents the non-linear relationship between the 2D coordinates for the egg outline projected on a 2D plane with a major axis about which the egg has rotational symmetry. There is no standard 2D egg curve defined yet but there are a number of different 2D egg curves proposed and studied by individuals. Köller [25] collected some 2D egg curve equations along with detailed descriptions. An equation of 2D egg curve proposed by [26] is given as follows:

(1) $${\left(X^{2}+Y^{2}\right)}^{2}=aX^{3}+(a-b)XY^{2}\;$$

where *a* and *b* are the egg shape parameters. Their ratio (*b*/*a*) governs how close the curve is to an actual egg’s outline [26]. The curve is shown in Figure 2. For $X\in [\begin{array}{cc} 0 & 0.05 \end{array}]$ and, *b*/*a* = 0.7. The parameter a can be treated as the physical length of the major axis (which coincides with the X-axis in Figure 2) of the egg, and *b* is normally 70% of *a* for a chicken egg (i.e., *b*/*a* = 0.7) according to an experiment performed by [26] based on 2D images of normal eggs.

**Figure 3:**
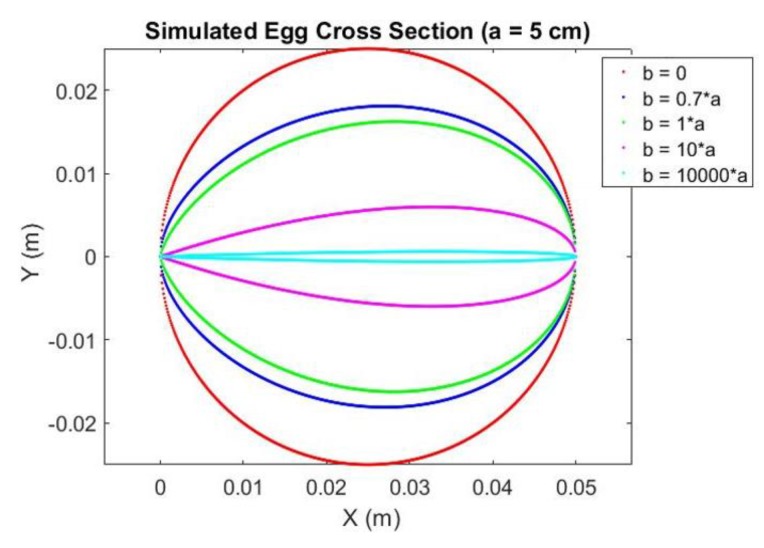
Simulated Egg Cross Section with Ratio of *b*/*a.*

Yamamoto [26] pointed out that a regular egg shape should satisfy *b*/*a* = 0.7. This can be visualized by the simulation of an egg cross section (which is a 2D egg) shown in Figure 3. When *a* is kept constant and *b* = 0, the cross section becomes a circle (sphere in 3D). As *b* gets larger the sphere tends to become an egg shape. When *b*/*a* is close to or equal to 0.7, the egg shape will become visually close to a regular egg. The increment of *b*/*a* will further shrink or flatten the egg as seen in Figure 3 that the egg cross section at *b* = 10,000*a* (cyan) only has a height of approximately 1 mm.

#### 2.3.2. Proposed 3D Egg Models

Yamamoto has provided a comprehensive review and proposed formulae of 2D and 3D eggs [26]. Yamamoto’s 3D egg equation is for an egg with its tip located in the origin, and it does not include position or orientation of the egg. Built on top of Yamamoto’s model, we proposed a set of novel 3D egg equations which can model an actual egg’s shape in a 3D object space along with its position and orientations. The proposed 3D egg equations are based on the principle that an egg shell can be treated as a cylinder in 3D space [27] with a varying radius along the egg’s major axis, while the radius is always zero at the two terminals of that axis. The varying radius is constrained by the 2D egg curve equations.

**Figure 4:**
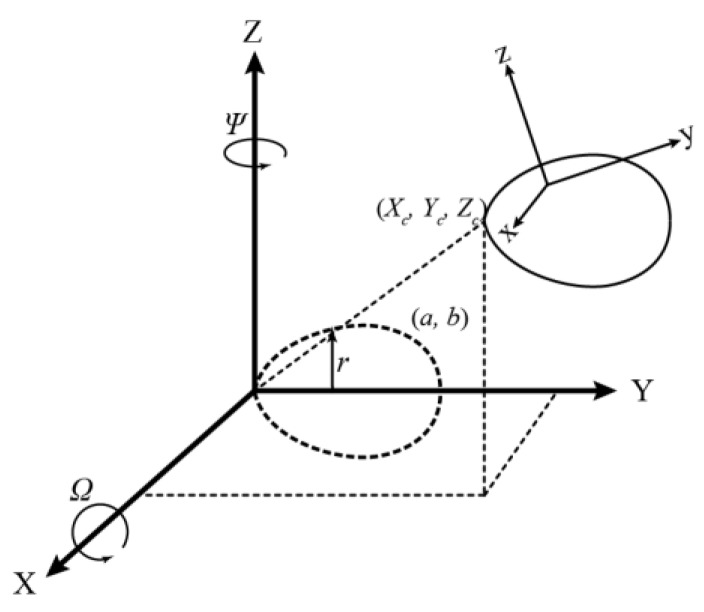
Proposed Egg Model Geometry (Model I).

**Figure 5:**
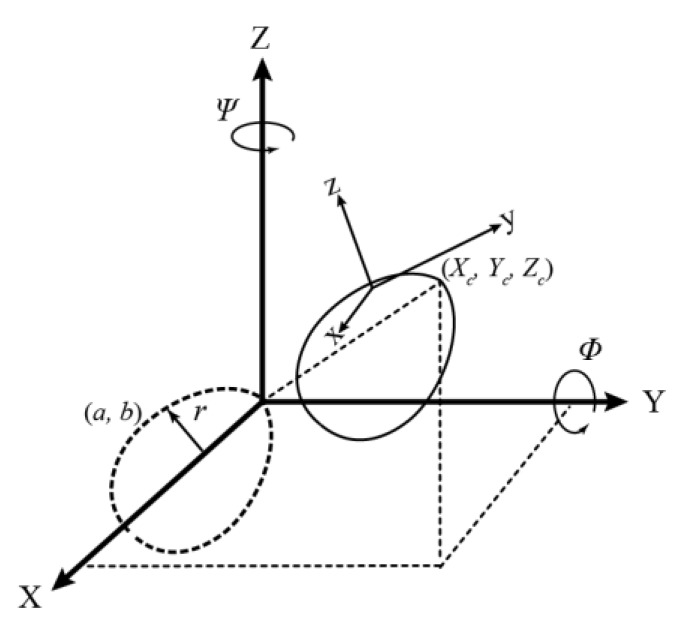
Proposed Egg Model Geometry (Model II).

Figure 4 shows the geometry of Model I, one of the two proposed 3D models for a horizontally aligned egg. The model transforms the egg in the sensor space (*x*, *y*, *z*) to a defined object space (*X*, *Y*, *Z*). The figure shows that the actual model parameters are the position (*X_c_*, *Y_c_*, *Z_c_*) of the tip of the egg, two rotation angles (*Ω* about the X-axis and *Ψ* about the Z-axis), and the two egg shape parameters *a* and *b* inherited from the 2D curve (Equation (1)). The parameter *r* is defined as a universal radius (perpendicular distance between any point of an egg shell to the Y-axis), but it is augmented with the *X* and *Z* coordinates so it is not needed to be estimated. As a result, any points on the egg shell can be transformed with the tip and two orientations to its nominal position (the dotted lines in Figure 4) which satisfies the 2D equation (Equation (1)) proposed by [26]. Model I is represented by Equations (2)–(4). If a major axis of a horizontal egg is closer (|*Ψ*| < 45°) to the X-axis, *Ω* will become less significant, in which case, another model defined as Model II (Equations (5)–(7)), with the rotation angle (*Φ*) about the Y (Figure 5) can be used:

(2) $${\left(Y^{2}+r^{2}\right)}^{2}=aY^{3}+(a-b)Yr^{2}$$

where:

(3) $$r^{2}=X^{2}+Z^{2}$$

and:

(4) $$\left(\begin{array}{c} X\\ Y\\ Z \end{array}\right)=R_{3}(\Psi )R_{1}(\Omega )\left[\left(\begin{array}{c} x\\ y\\ z \end{array}\right)-\left(\begin{array}{c} X_{c}\\ Y_{c}\\ Z_{c} \end{array}\right)\right]$$

and **R**_1_ and **R**_3_ are the rotation matrices about the X and Z-axes, respectively.

Similarly, Model II, with the egg’s major axis coincides with the X-axis is given by:

(5) $${\left(X^{2}+r^{2}\right)}^{2}=aX^{3}+(a-b)Xr^{2}:$$

where:

(6) $$r^{2}=Y^{2}+Z^{2}$$

and:

(7) $$\left(\begin{array}{c} X\\ Y\\ Z \end{array}\right)=R_{3}(\Psi )R_{2}(\Phi )\left[\left(\begin{array}{c} x\\ y\\ z \end{array}\right)-\left(\begin{array}{c} X_{c}\\ Y_{c}\\ Z_{c} \end{array}\right)\right]$$

and **R**_2_ are the rotation matrices about the Y-axes.

**Figure 6:**
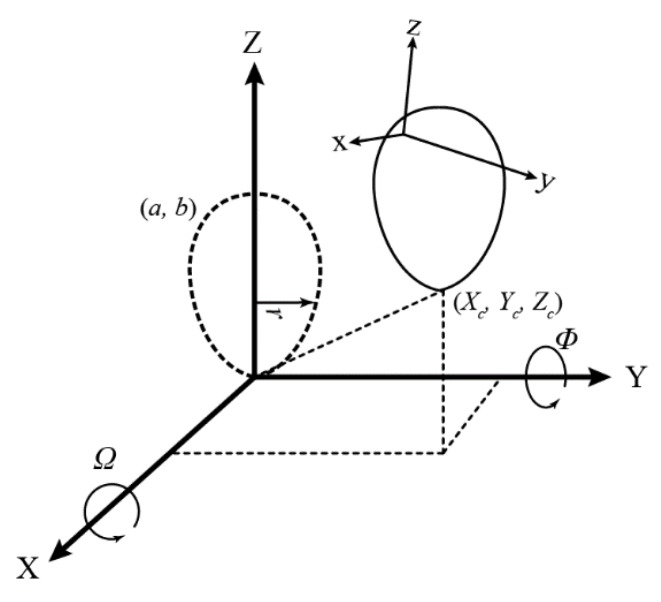
Proposed Egg Model Geometry (Model III).

Since the egg naturally lies horizontally on any flat surfaces due to the gravity, only the two above horizontal egg models (Models I and II) are used in this research. However, the vertical egg model, Model III (Equations (8)–(10)) is also presented herein for completeness (when 45° < |*Ω*| < 135° or 45° < |*Φ*|< 135°). Its geometry is depicted in Figure 6. 

Model III for eggs in a vertical direction is given by:

(8) $${\left(Z^{2}+r^{2}\right)}^{2}=aZ^{3}+(a-b)Zr^{2}$$

where:

(9) $$r^{2}=X^{2}+Y^{2}$$

and:

(10) $$\left(\begin{array}{c} X\\ Y\\ Z \end{array}\right)=R_{2}(\Phi )R_{1}(\Omega )\left[\left(\begin{array}{c} x\\ y\\ z \end{array}\right)-\left(\begin{array}{c} X_{c}\\ Y_{c}\\ Z_{c} \end{array}\right)\right]$$

**Figure 7:**
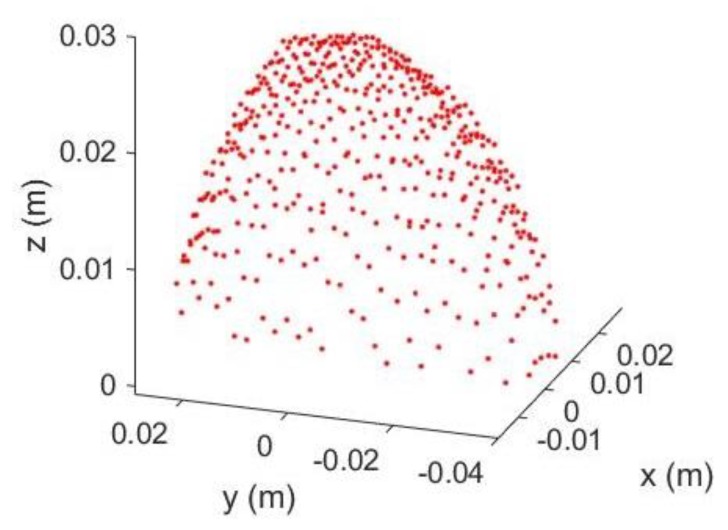
Shear-like Egg Point Cloud.

**Figure 8:**
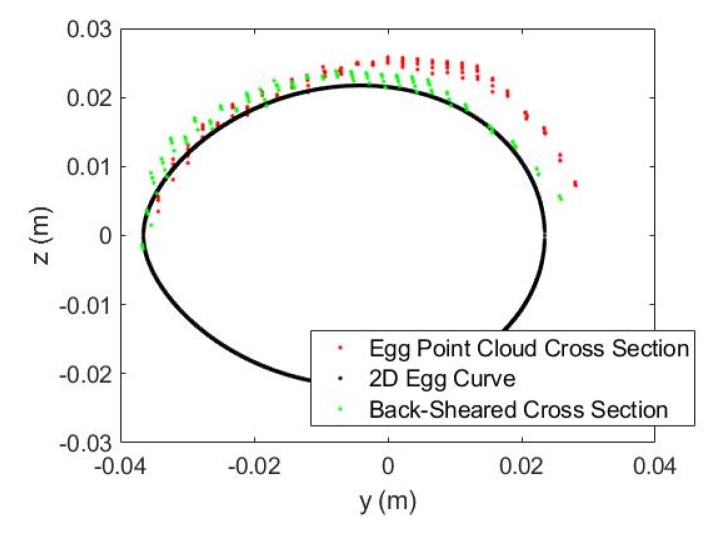
Shear-like Egg Point Cloud Cross Section and its Back-sheared.

The Kinect 2.0’s depth measurement incurs different types of errors [17,28] which deforms the acquired 3D point clouds of the curved egg surface and thus seriously deteriorate the estimated egg volume. Some systematic errors can be reduced by calibration with a target field [29,30,31] but this is usually not a straightforward procedure for end-users. Additionally, some of the errors are time-varying in nature (e.g., temperature dependent errors, [28]), which cannot readily be removed by performing the calibration. As a result, an alternative approach that can reduce the errors adhered to the egg point clouds without recourse to calibration is preferred. After observing “shear-like” patterns in the captured egg point clouds (e.g., Figure 6), we propose the inclusion of conventional 3D shear parameters [32], *Sh*_x_ and *Sh*_y_ for the X and Y directions (Equation (11)), respectively, in the models and after the egg is transformed to the object space to absorb the measurement errors of the egg shell. The back-sheared coordinates ($X^{\prime },Y^{\prime }$) are used to replace the corresponding coordinates (X,Y) in Model I and II when the shear parameters (the negative sign indicates the back-shear) are considered in the models. A shear-like egg point cloud collected by the Kinect is shown in Figure 7. Its cross section shown in Figure 8 is sheared (red) and it can be back-sheared by including the shear parameters:

(11) $$\{\begin{array}{c} X^{\prime }=X-Sh_{x}Z\\ Y^{\prime }=Y-Sh_{y}Z \end{array}$$

Depending on the errors at a particular instance and conditions (e.g., camera temperature), the models with the shear parameters may not be applicable for all cases, and therefore sometimes the original models without the shear parameters should be kept to deliver higher fitting accuracy. To determine when the shear parameters should be included, the sum of the squares of the residual of the fittings and the ratio of the estimated egg curve parameters (*b*/*a*) can both be potential indicators. More specifically, the shear parameters should be included in the models when the sum of the squares of the residuals is reduced compared to the fittings without the shear parameters. Secondly, the *b*/*a* ratio should fall within ±10% of 0.7. This criterion guarantees the estimated egg shape to be realistic. Further discussion about the ratio of *b*/*a* is given in Section 2.4.

### 2.4. Volume Estimation Method

Since the parameters and observations are not separable in the models, the egg model parameters are solved using the Gauss-Helmert least-squares adjustment model [33]. The the linearized adjustment model is given by:

(12) $$A\hat{\delta }+B\hat{v}+w=0$$

where $\hat{\delta }$ is the correction vector for the $\vec{X}=\left[\begin{array}{ccccccc} \Omega & \Psi & X_{c} & Y_{c} & Z_{c} & a & b \end{array}\right]$ or $\vec{X}=\left[\begin{array}{ccccccccc} \Omega & \Psi & X_{c} & Y_{c} & Z_{c} & a & b & Sh_{x} & Sh_{y} \end{array}\right]$ for Model I; **A** is the design matrix of partial derivatives of the functional model (e.g., Model I) with respect to the parameters; **B** is the design matrix of partial derivatives of the functional model with respect to the Kinect’s observations (*x y z*); $\hat{v}$ is the vector of residuals; and *w* is the misclosure vector.

Once the egg shape parameters *a* and *b*, are estimated, the volume (V) of the eggs can be computed as follows [26]:

(13) $$V=\pi \int_{0}^{a}r^{2}d\hat{Y}=\frac{\pi }{2}\left\{\frac{a}{6b}{\left(a+b\right)}^{3}+\frac{1}{60b^{2}}\left[{\left(a-b\right)}^{5}-{\left(a+b\right)}^{5}\right]-\frac{1}{6}a^{3}-\frac{1}{2}a^{2}b\right\}$$

which is a function of *a* and *b* only, and is independent of the position, orientation and shear parameters. The standard deviation of the volume can be computed as follow according the law of error propagation [34]:

(14) $$\sigma_{V}=\sqrt{{\left(\frac{\partial V}{\partial a}\right)}^{2}\sigma_{a}^{2}+{\left(\frac{\partial V}{\partial b}\right)}^{2}\sigma_{b}^{2}+2\frac{\partial V}{\partial a}\frac{\partial V}{\partial b}\sigma_{ab}}$$

where *σ_a_* and *σ_b_* are the variance obtained from the least-squares estimation for egg shape parameter *a* and *b*, respectively, and *σ_ab_* is the covariance of *a* and *b*.

**Figure 9:**
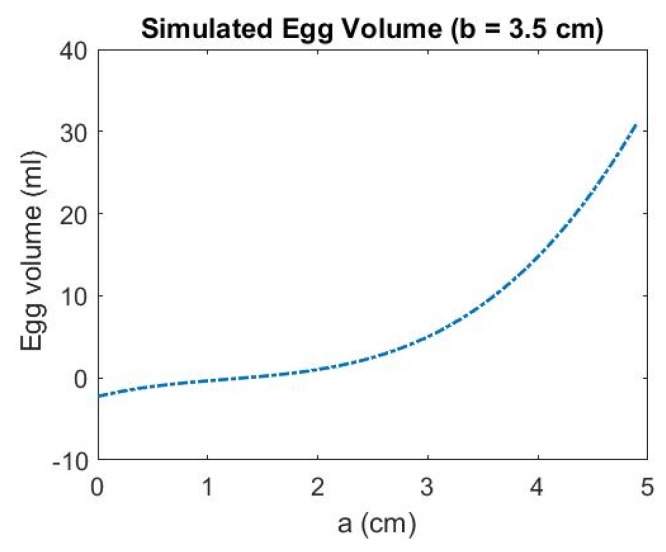
Simulated Egg Volume with Ascending *a* (*b* is kept constant).

**Figure 10:**
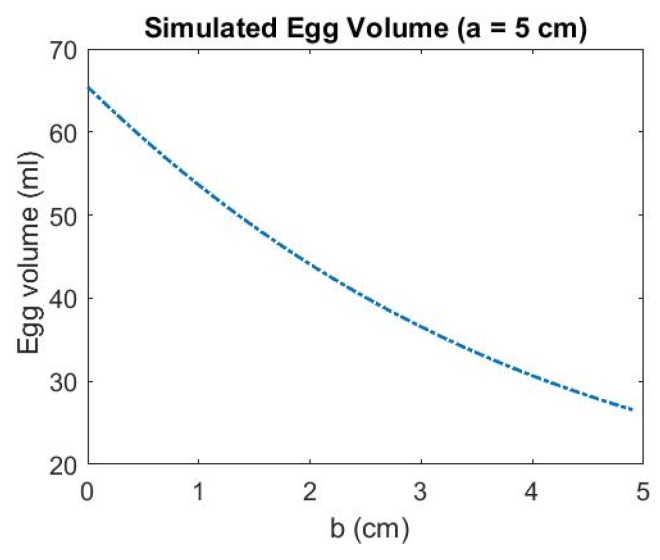
Simulated Egg Volume with Ascending *b* (*a* is kept constant).

Since the volume is only dependent of *a* and *b*, it is worth investigating how the change of the parameters affects the estimated volume. The estimated egg volumes are simulated with the ascending a while b is kept constant and vice versa. The resulting volumes are shown in Figure 9 and Figure 10, respectively. It can be seen that when the egg is larger (with longer major axis, i.e., *a* is larger), the accuracy of the estimated parameter a become more significant (the rate of change of the volume with respect to *a* is larger when a is larger than 3 cm) than parameter b to the estimated volume. Therefore, when the egg is larger, the accuracy of *a* is more significant than b for the volume estimation.

### 2.5. Proposed Processing Algorithm

#### 2.5.1. Automatic Egg Extraction from Raw Point Cloud

The depth images or point cloud (.xyz file) and the infrared intensity images (.png files) of the entire scene were processed to obtain only the egg shell point cloud for further analysis. First, the values from the infrared intensity file were assigned to each point stored in the .xyz file. Since the egg is close to the center of the image, a window with a specific size (e.g., 30 cm × 30 cm) was used to extract the point cloud near the center, which only contains the egg and part of the stage (assume a dark stage). Then, the conventional Otsu method [35] was used to separate the egg from the stage based on the assigned infrared intensity values. Once the egg point cloud is obtained, outliers are filtered out by using a robust surface fitting technique [36] such that the impact of outliers for the subsequent egg model fitting is minimized.

#### 2.5.2. Initial Value Computation Algorithm for the Models

**Figure 11:**
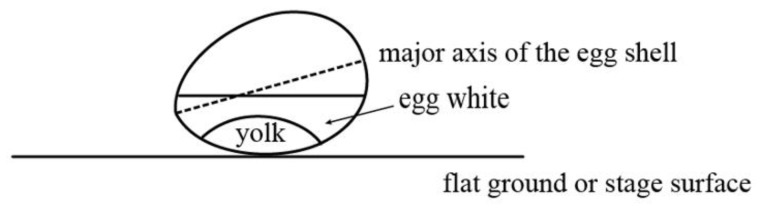
Tilted Egg Geometry and the Kinect Position.

**Figure 12:**
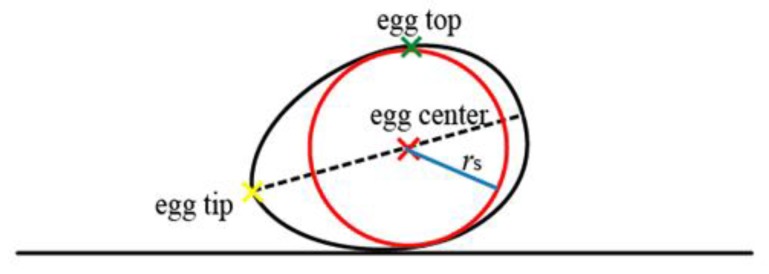
Theoretical Best Fit Sphere (Red) and the defined Egg Center.

Since the egg model is a high order (4th) of polynomial, an accurate set of initial values of model parameters is essential for the least-squares estimation. As seen in Figure 11, the egg is always tilted relative to the ground (or stage) and the Kinect (levelled) due to the gravity experienced by the yolk. Therefore, nominal values of the orientation (e.g., 0° for vertical orientation) cannot be used as the initial values of the parameters. On the other hand, only about a half of the egg can be captured by the Kinect, therefore it can be seen that the egg is symmetric in XY direction but not in the Z direction. The orientation of the major axis has to be known in order to estimate the initial rotation angles, so a well-defined egg center is needed (Figure 12).

**Figure 13:**
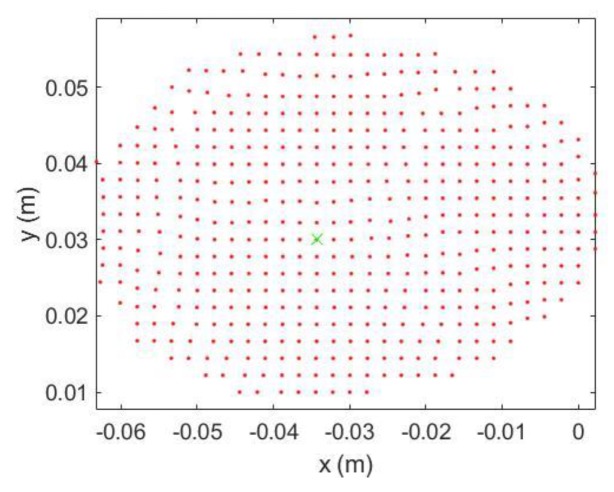
Egg Point Cloud 2D view from the Top.

An egg shell point cloud processing algorithm is developed to compute the initial parameters, and the main steps are listed as follows:

*Step 1*: Find the egg top point (green cross in Figure 13) by searching the point with the minimum point distance (depth). Then, treat the egg as 2D structure and only use the XY coordinates. Translate XY coordinates of the egg points with the XY coordinates of the egg top (X_top_, Y_top_).

*Step 2*: Estimate a rotation angle (θ) which results in a minimum value of max(Y’) − min(Y’) with [0 180°] using the golden section search and parabolic interpolation [37], where Y’ can be computed using Equation (15). The algorithm decides which model (Model I or II) will be used for the fitting based on estimated θ. Hereon, Model II is assumed to be used:

(15) $$\left(\begin{array}{c} X^{\prime }\\ Y^{\prime } \end{array}\right)=\left(\begin{array}{cc} \cos\left(\theta \right) & -\sin\left(\theta \right)\\ \sin\left(\theta \right) & \cos\left(\theta \right) \end{array}\right)\left(\begin{array}{c} X-X_{top}\\ Y-Y_{top} \end{array}\right)$$

*Step 3*: After applying the transformation (Equation (15)), the major axis of the 2D egg is almost parallel with the X-axis. Therefore, compute the approximate radius, *r*_s_ (blue in Figure 12) of the best fit sphere (red in Figure 12) using the egg point cloud as [max(Y’) − min(Y’)]/2.

**Figure 14:**
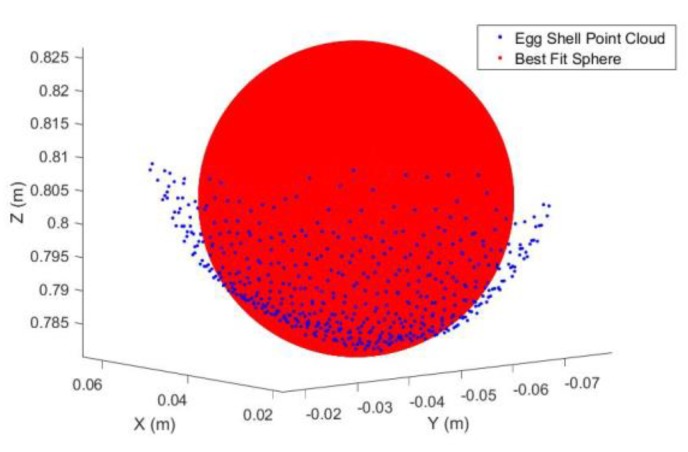
Best Fit Sphere to the Egg Shell Point Cloud.

*Step 4*: Perform sphere least-squares fitting with the constrained known *r*_s_ with the original 3D egg shell point to compute the egg center (Figure 12). An actual sphere fitting to the egg point cloud is shown in Figure 14. If *r*_s_ is not constrained in the fitting, the fitted sphere will be bigger as its diameter will become the entire length of the major axis of the egg.

*Step 5*: Repeat Step 2 to translate the egg point cloud using the fitted sphere center instead of the egg top as the egg center is more accurate representation of the egg’s centroid. After egg point cloud is transformed, find the egg tip (Figure 12) by searching a point with the minimum distance from the X-axis but with maximum distance from the egg center.

*Step 6*: Once the two points (3D coordinates of the egg tip and egg center) are found, compute the straight line connecting these points which coincides with the egg’s major axis. Translate the line and egg tip to the origin and compute its orientation using basic trigonometry. The orientation of the line (major axis) is equivalent to the orientation of the egg. Finally, compute the initial value of a by max(X’) − min(X’). Then, compute the initial value of *b* using the formula, *b* = 0.7*a* [26].

## 3. Experiments

The experiments were mainly conducted at the calibration room of the Department of Geomatics Engineering at the University of Calgary on 9 June 2017 and 28 June 2017. A few sets of independent datasets were collected from eight different eggs, namely Egg I to VIII in this paper, with slightly different sizes. The Kinect was set aside for 30 min warm-up [30] before the point clouds were captured.

### 3.1. Experimental Setup

**Figure 15:**
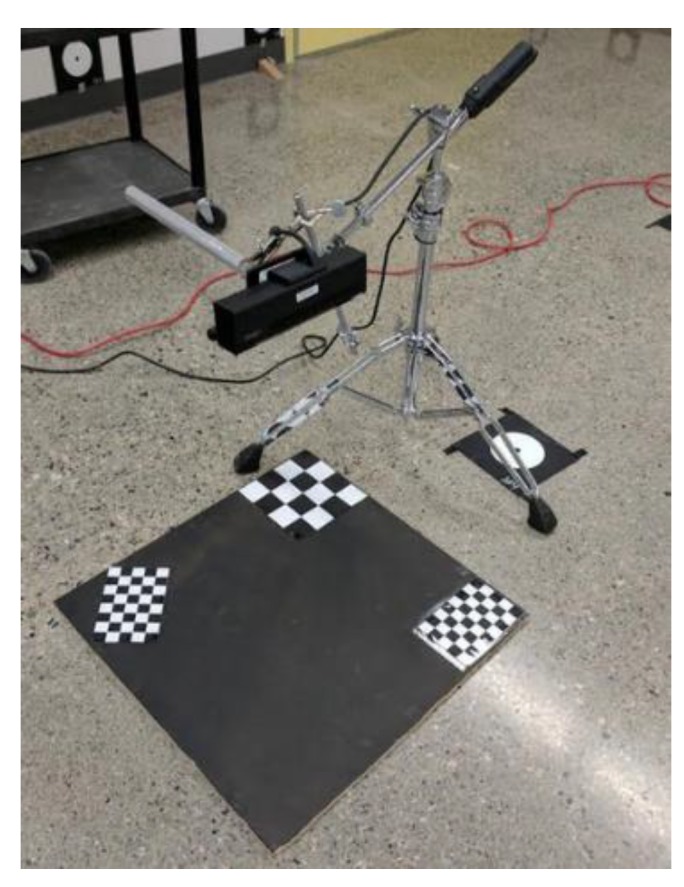
The Experimental Step with a specifically designed tripod.

**Figure 16:**
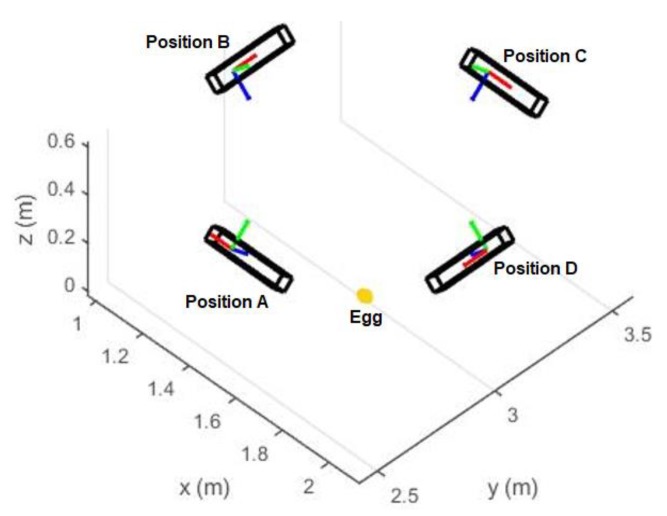
Four different Kinect Positions. The Kinect is tilted 45º with respect to the egg. The x, y and z sensors of the sensor space is represented by red, green, and blue color, respectively.

**Figure 17:**
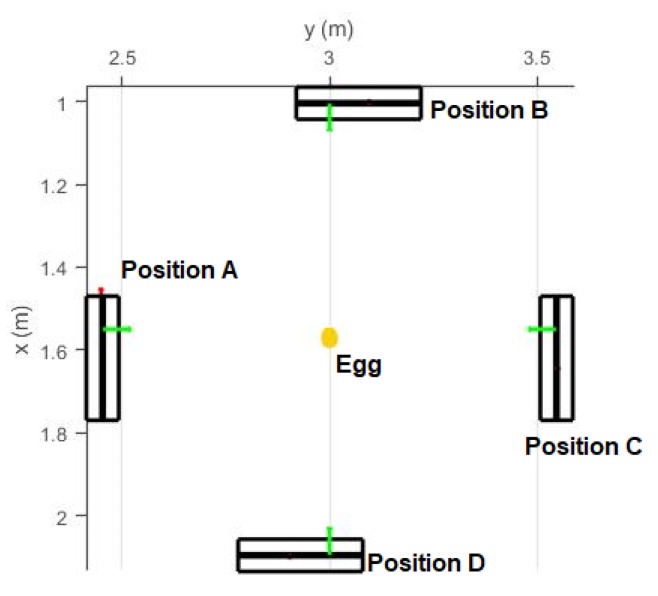
Four different Kinect Positions (Bird Eye view of Figure 16).

Four different sets of experiment were performed (Table 1): The first experiment was conducted at different Kinect-to-Egg distances for two eggs (Eggs I and II). The distance was varied using a specifically designed tripod with an adjustable arm (Figure 15). The stage for the egg is a wood board with a checkerboard pattern to perform a study of the point cloud registration (no registration results are discussed in this paper as this is defined as out of scope). The egg was placed at nadir of the Kinect, and roughly faced opposite to the center of the infrared sensor of the Kinect. The Kinect-to-Egg distances were adjusted from 60 cm to 90 cm at every two-centimeter interval. The aim of this experiment is to estimate the optimal Kinect-to-Egg distance that can result in a point cloud with the least error. For the second set of experiments, four different Kinect positions (Figure 16 and Figure 17) were used to capture two other eggs (Eggs III and IV), the Kinect was approximately tilted by 45°. The egg is isotropic with respect to the Kinect as long as it faces to the Kinect’s centre and its major axis is either parallel or perpendicular to the Z-axis of the Kinect. In other words, the same point clouds (same number and distribution of points) of an egg can be obtained at different orientations to the Kinect at a fixed Kinect-to-Egg distance as long as the egg faces the Kinect centre and its major axis is either parallel or perpendicular to the Z-axis of the Kinect. However, different positions of the Kinect can impact the number and the distribution of points for the collected egg point clouds. The aim of this experiment is to verify if the method can be used placing the Kinect at different locations. The third experiment is performed to examine if the method works well with other bird eggs. Duck and quail eggs were used (Eggs V and VI) in this experiment. In the fourth experiment, a Faro Focus^3D^ laser scanner (Faro Technologies Inc., Lake Mary, FL, USA) was used to scan the eggs (Eggs VII and VIII) to verify if the proposed method also works properly with the point cloud obtained from another 3D sensor. The results from the Faro scanner can also serve as another set of volume references. Details of the eggs used in the experiment are tabulated in Table 1.

### 3.2. Assumption

The assumptions made in this work are summarized as the follows.

1. The Kinect-to-Egg distances were initially measured manually by using tapes, and the adjusted distances was performed according to markers found on the tripod. The overall measurement error is assumed to be ±1 cm.
2. The reference egg volumes were mainly obtained using the water displacement method which is based on the Archimedes Principle. The precision for the water displacement was assumed to be ±2 mL for all the volumes.
3. The precision of the Kinect’s captured point cloud for the eggs is set to be ±1 mm for the least-squares fitting.

## 4. Results and Analysis

### 4.1. Accuracy versus Capturing Distance

**Figure 18:**
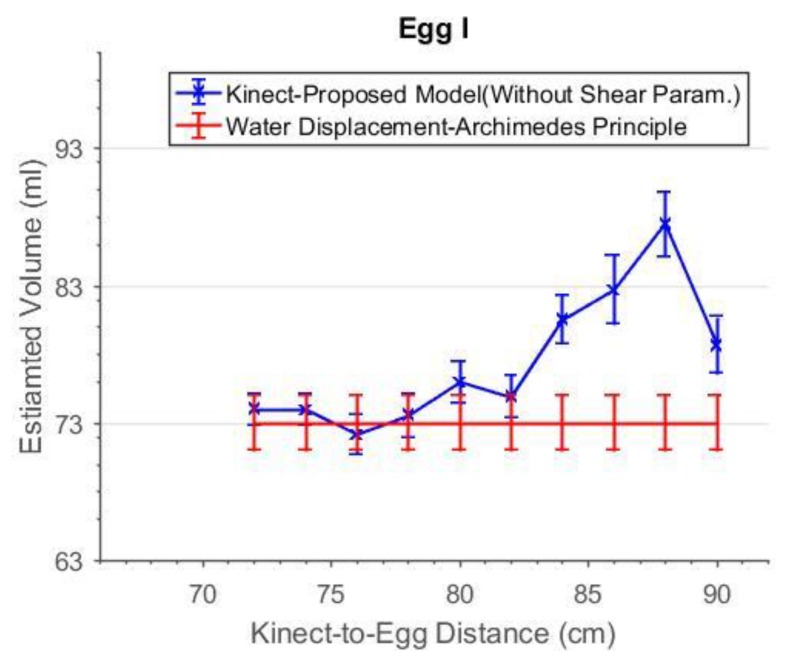
The estimated volume without shear parameters versus capturing distance for Egg I.

**Figure 19:**
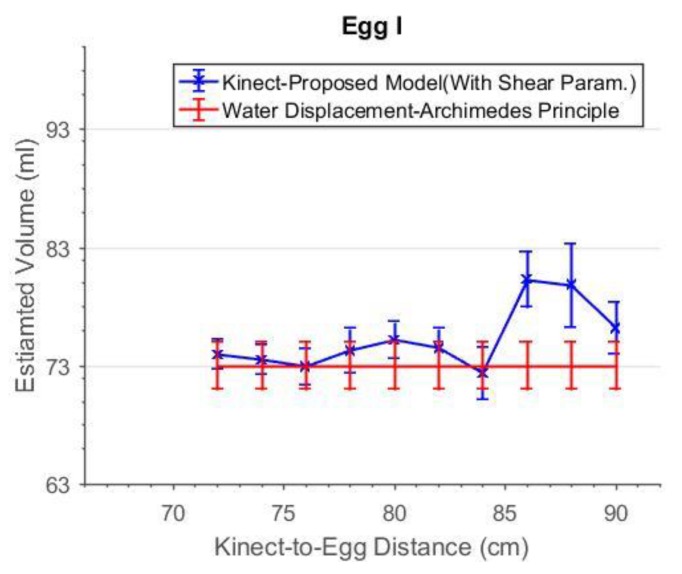
The estimated volume with shear parameters versus capturing distance for Egg I.

The range errors of the Kinect increase with distances in general [17]. However, if the capturing distance is short enough, scattering resulted from background object [30] occur which will negatively distort the point cloud. Therefore, a distance which can allow the Kinect to capture the most accurate egg point cloud should first be quantified. At each distance, the egg volume was estimated and compared to the volume reference obtain from the water displacement experiment (the Archimedes Principle). The estimated volume of Egg I with and without the shear parameters are shown in Figure 18 and Figure 19, respectively. There was no estimated volume for distances less than 72 cm as the egg point clouds were extremely distorted due to the scattering and so the least-squares fitting did not converge to deliver a solution. From Figure 18, it is known that the estimated volume of Egg I has the best accuracy from 72 cm to 78 cm.

**Figure 20:**
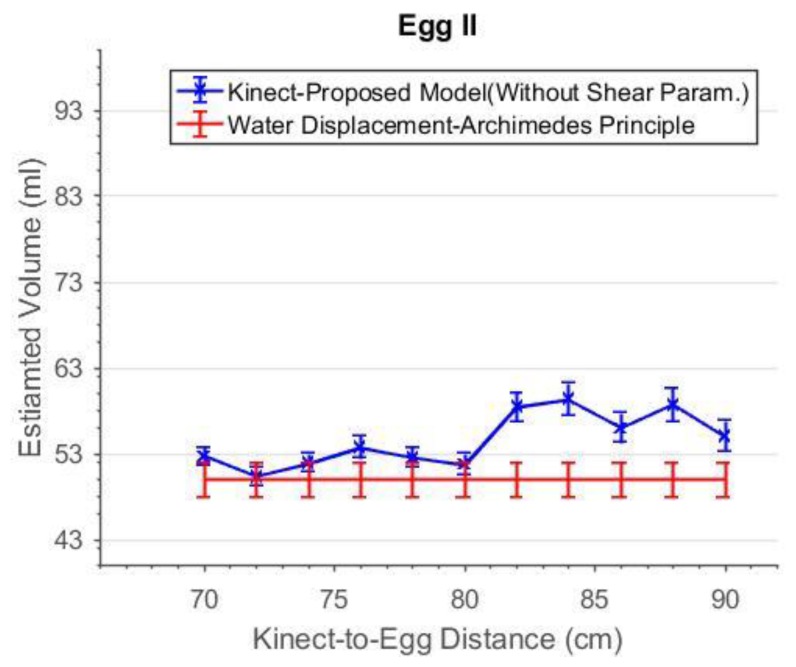
The estimated volume without shear parameters versus capturing distance for Egg II.

**Figure 21:**
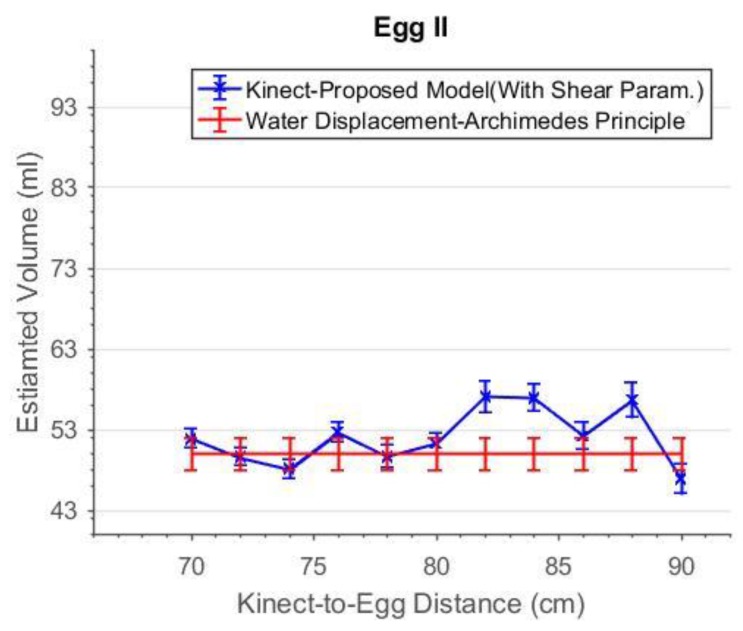
The estimated volume with shear parameters versus capturing distance for Egg II.

**Figure 22:**
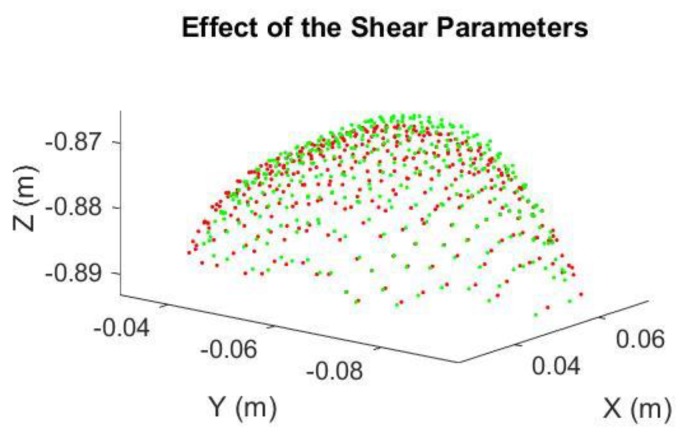
The Egg Point Cloud: Original (Red), Shear Corrected (Green).

**Figure 23:**
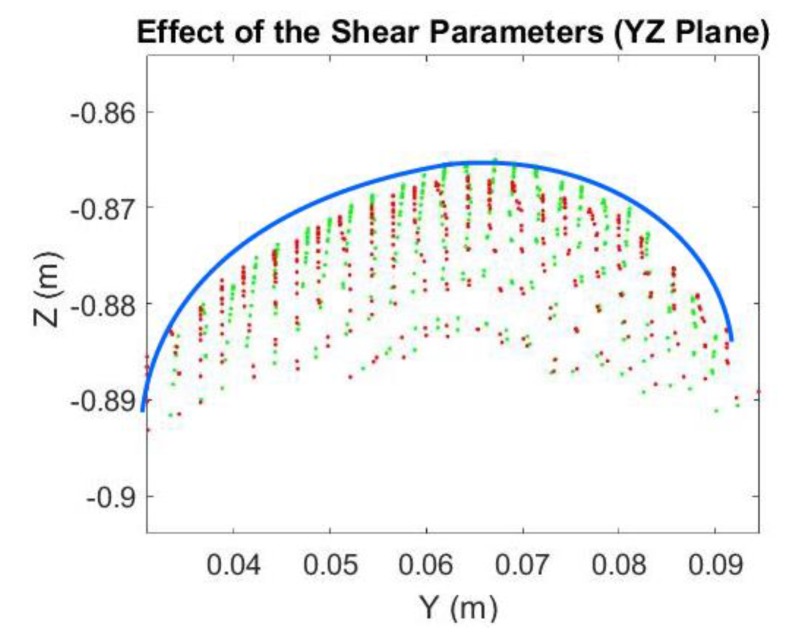
The Egg Point Cloud (YZ Plane): Original (Red), Back-Shear Corrected (Green), Best Fit Egg Model (Blue).

As can be seen in Figure 19, the accuracies of the estimated volume from 76 cm to 84 cm were improved after the shear parameters were applied. The degraded precisions compared to the “shear-free” estimations are due to the reduction in degree of freedom as two more additional parameters are solved. From 72 cm to 84 cm, the estimated volumes match the reference better when the shear parameters are included in the model. The experiment was repeated with a smaller egg, Egg II. The estimated volume of Egg II without and with the shear parameters are shown in Figure 20 and Figure 21, respectively. It can be seen that the best estimated volumes were yielded from 70 cm to 80 cm. The largest volume error of the estimated volume of Egg I was found at 88 cm. This error was reduced by 5 mL (approximately 6%) after the shear parameters were included in the model. For visualization, the effects of the shear parameters for Egg I (at 88 cm) are shown in Figure 22 and Figure 23. Overall, the egg volume can be estimated accurately at a Kinect-to-Egg range of approximately 70 to 78 cm. A Kinect-to-Egg distance close to 74 cm is recommended to obtain the best volume accuracy. In general, the results indicate that including the shear parameters will improve the volume accuracy.

### 4.2. Accuracy versus Capturing Positions

Different Kinect positions will affect the number of point and also the point distribution, and this will seriously affect the quality of the geometric fitting. In this section, the volume estimation results from the four different Kinect Positions A, B, C and D shown in Figure 16 and Figure 17 are analyzed. When the major axis is perpendicular to the z-axis of the Kinect (Positions A and C), more points can be captured along the major axis of the egg but less points can be captured along the z-direction of the egg (perpendicular to the major axis of the egg). This condition reverses for Positions B and D. Table 2 and Table 3 show the estimated volumes (using the models without and with the shear parameters independently) of two eggs (Egg III and Egg IV) at the four different positions. The standard deviation of the volume was derived using Equation (14) with the precision of *a* and *b* obtained from the adjustment. From the tables, it can be seen that the estimated volumes are comparable with the reference volumes at Positions A and C but are deviating significantly from those at Positions B and D. This is because Positions A and C allow the Kinect to capture more points along the egg’s major axis. More observations along the major axis of the egg help determine the shape of the egg (the parameter *a* and *b*) as the egg curve is defined in such a way that the perpendicular distance between the egg shell and the major axis vary along the major axis. Also, from the tables, it can be seen that the parameter *a* is also more accurately estimated from Positions A and C. This results in more accurate volume estimation. The accuracy of the parameter *a* has proved to be more significant than that of parameter *b* for larger eggs (eggs with major axis greater than 3 cm, which holds for regular chicken egg) based on the simulation (Figure 9).

**Figure 24:**
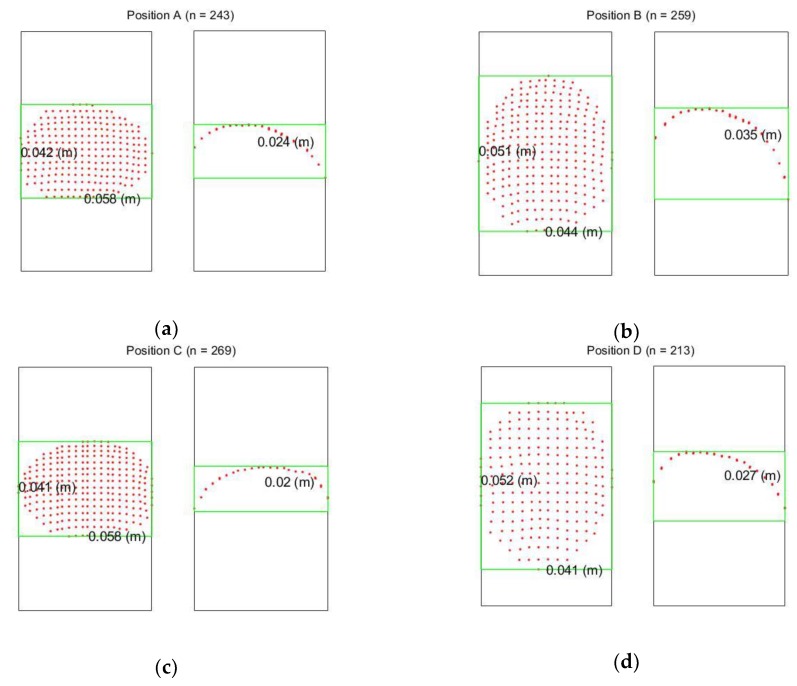
The Nominal Dimension of the Point Cloud for Egg III at different positions: (**a**) Position A; (**b**) Position B; (**c**) Position C; (**d**) Position D; Left for the bird eye view while right is the cross-section along the major axis. n is the number of points captured.

Figure 24 shows the nominal dimensions of the point cloud of Egg III at different positions. The nominal dimensions of the egg shown in the figure were computed by using the maximum/minimum of the X, Y and Z directions of the captured egg point cloud (half of the egg). By comparing the egg point cloud dimensions obtained from Positions A and C, to those obtained from Positions B and D, it is known that the Positions A/C capture a larger portion of the egg surface along the major axis and therefore yields more accurate estimation of the egg shape parameters and thus the volumes. It can be observed from Table 2 and Table 3, and Figure 24 that more point observations along the egg’s z direction do not benefit the estimation of the egg shape parameters and volume. 

Similar to the above section, the results shown in Table 2 and Table 3 also indicate that the shear compensation improves the volume accuracy but the least-square estimation for the model with the shear parameters did not converge for the captured data at Positions B and D due to lack of observations along the major axis. Overall, the egg volume estimation is promising as long as the major axis of the egg is perpendicular to the Kinect allowing a more complete capture of the eggs surface.

### 4.3. Different Types of Eggs

It can be useful to investigate whether the proposed method can be generalized for other bird eggs. Table 4 shows the estimated volumes of a duck egg (Egg V) and a quail egg (Egg VI). The results indicate that the proposed method works well with these two egg types. Only 78 points were captured for the quail egg, while there were about 280 points for the duck egg, which means the degrees of freedom are much lower for the quail egg as the numbers of the estimated parameters for the least-squares fittings are the same for both eggs. This is reason why the precision for the quail egg is much lower compared to the estimated volume, which is approximately 21% of the estimated value (only approximately 4% for the duck egg).

### 4.4. Egg Point Cloud from Laser Scanner

Laser scanners are often considered as references for other optical sensors with higher measurement noises [38]. The proposed method was tested with the point cloud captured by a Faro Focus^3D^ laser scanner and compared with the result obtained from the Kinect. Table 5 shows the results of the estimated volume without the shear parameters for two eggs (Eggs VII and VIII). It can be seen that the results obtained from both Kinect and the Faro are very comparable. Nevertheless, preliminary results suggest that larger angle of incidence and scanning distance will reduce the estimated volume accuracy for Faro’s point cloud but the performance analysis of the egg volume estimation for laser scanning is not the focus of this work, thus it is not discussed herein. A triangulation-based scanner, e.g., Minolta Vivid 9i, may produce even more accurate results but further experimental work is needed.

### 4.5. Final Accuracy

The developed prototype focuses on chicken egg volume estimation. Table 6 shows the summary of the volume accuracy of the chicken eggs. It can be seen that up to approximately 98% accuracy was achieved, and in average 93.3% of the accuracy was recorded for our proposed cost-efficient prototype. The recommended configuration for the system is to place the egg approximately 74 cm from the Kinect, and its major axis should always be perpendicular to the Kinect’s z axis.

## 5. Conclusions

In this paper, we present a cost-effective egg metrological system prototype which can deliver accurate volume measurements with minimal human intervention. The system is equipped with the Microsoft Kinect 2.0 which captures 3D point cloud of the eggshell. For our developed processing algorithm, the point clouds are modelled with the proposed 3D egg equations. The egg shape parameters are estimated along with position/orientation parameters using the least-squares method to further compute the egg’s volume. The method requires only a point cloud of approximately one half of the eggshell and therefore point cloud registration is not needed. As a result, the method is highly automatic and can be readily transformed to a fully real-time egg volume measurement system. The proposed method was verified with the reference obtained from water displacement (the Archimedes Principle) and laser scanner point clouds. The method not only works well with chicken eggs, but also with other bird eggs such as duck and quail eggs. Some working conditions such as optimal capturing distance for the system were examined. The results suggested that the proposed method can deliver an average estimated volume accuracy of 93.3% when compared to the water displacement references. The prototype has high potential to be further improved to form a real-time egg volume measurement system which can estimate volumes of groups of eggs being transferred on a conveyor belt. Also, the developed algorithm can be adopted to newer the range cameras in the future. 

## Figures and Tables

**Table 1:** Summary of the Egg used in the Experiments.

Egg Label	Egg Type	Approximate Dimensions L (cm) × r (cm)	Experiment Description	Estimated Results
I	Chicken	6 × 2	Different Kinect-to-Egg Distances	Section 4.1
II	Chicken	5.5 × 1.75		
III	Chicken	6 × 2	Different Kinect Position	Section 4.2
IV	Chicken	5.5 × 2		
V	Duck	7 × 2.25	Different Bird Eggs	Section 4.3
VI	Quail	3 × 1		
VII	Chicken	6 × 2	With Laser Scanner	Section 4.4
VIII	Chicken	5.5 × 1.75		

**Table 2:** Estimated Egg Volumes of Egg III.

Capturing Position	Without Shear Param.		With Shear Param.		Ref. Vol. (mL)
	Vol. (mL)	Est. Egg Shape Param. |a|, |b| (cm)	Vol. (mL)	Est. Egg Shape Param. |a|, |b| (cm)	
A	68.69 ± 2.65	6.50 ± 0.15 5.04 ± 0.51	67.24 ± 9.54	6.17 ± 0.28 3.84 ± 0.82	66 ± 2.0
B	54.70 ± 1.86	5.70 ± 0.11 3.37 ± 0.32	No Solutions	No Solutions	
C	63.72 ± 1.80	6.14 ± 0.06 4.09 ± 0.24	66.66 ± 4.08	6.07 ± 0.13 3.54 ± 0.29	
D	54.03 ± 2.12	5.45 ± 0.18 5.78 ± 0.68	No Solutions	No Solutions	

**Table 3:** Estimated Egg Volumes of Egg IV.

Capturing Positon	Without Shear Param.		With Shear Param.		Ref. Vol. (mL)
	Vol. (mL)	Est. Egg Shape Param. |a|, |b| (cm)	Vol. (mL)	Est. Egg Shape Param. |a|, |b| (cm)	
A	55.04 ± 2.30	5.68 ± 0.07 3.24 ± 0.24	53.25 ± 6.62	5.63 ± 0.26 3.29 ± 0.43	51 ± 2.0
B	45.44 ± 2.28	5.16 ± 0.80 2.41 ± 0.25	No Solutions	No Solutions	
C	54.11 ± 3.62	5.57 ± 0.08 2.91 ± 0.27	52.31 ± 6.47	5.70 ± 0.25 3.62 ± 0.45	
D	42.58 ± 3.62	5.41 ± 0.14 3.75 ± 0.46	No Solutions	No Solutions	

**Table 4:** Estimated Egg Volumes of Egg V and VI.

Egg Type	Vol. (mL)	Est. Egg Shape Param. |a|, |b| (cm)	Ref. Vol. (mL)
Duck Egg (Egg V)	74.85 ± 2.12	6.76 ± 0.05 5.49 ± 0.23	74 ± 2.0
Quail Egg (Egg VI)	11.78 ± 1.74	3.23 ± 0.18 1.33 ± 0.32	11 ± 2.0

**Table 5:** Estimated Egg Volumes of Egg VII and VIII.

Capturing Position	Kinect		Faro Focus^3D^		Ref. Vol. (mL)
	Vol. (mL)	Est. Egg Shape Param. |a|, |b| (cm)	Vol. (mL)	Est. Egg Shape Param. |a|, |b| (cm)	
VII	64.96 ± 2.86	6.18 ± 0.18 4.14 ± 0.34	65.74 ± 4.08	6.44 ± 0.23 5.15 ± 0.51	66 ± 2.0
VIII	50.31 ± 1.78	5.84 ± 0.06 4.49 ± 0.24	48.56 ± 3.07	5.55 ± 0.12 3.51 ± 0.62	51 ± 2.0

**Table 6:** Summary of the Volume Accuracy of the Chicken Eggs.

Egg Label	With Shear Param.	Est Vol. (mL)	Ref. Vol. (mL)	Accuracy (%)
I	Yes	73.07 ± 2.25	73 ± 2	93.92
II	Yes	50.02 ± 2.48	50 ± 2	95.00
III	Yes	66.66 ± 4.08	66 ± 2	92.82
IV	Yes	52.31 ± 6.47	51 ± 2	84.75
VII	No	64.96 ± 2.86	66 ± 2	95.44
VIII	No	50.31 ± 1.78	51 ± 2	97.86
Mean				93.30
